# Trends and intellectual property landscape in soybean breeding: a patent and paper Bibliometric analysis

**DOI:** 10.1080/21645698.2026.2662120

**Published:** 2026-05-05

**Authors:** Maria Fernanda Sua-Rojas, Daniel Neris, Marcelo Menossi

**Affiliations:** a Laboratório de Genoma Funcional, Departamento de Genética, Evolução, Microbiologia e Imunologia, Instituto de Biologia, Universidade Estadual de Campinas, UNICAMP, Campinas, Brazil; b Centro de Melhoramento Molecular de Plantas (CeM^2^P), Universidade Estadual de Campinas, UNICAMP, Campinas, Brazil

**Keywords:** Bibliometric analysis, Glycine max, intellectual property, patents, research papers, soybean breeding

## Abstract

Soybean [*Glycine max* (L.) Merr.] is one of the most important crops globally. Researchers have focused on enhancing soybean through genetic modification, leading to an extensive accumulation of publications and patents. Despite this, a comprehensive exploration of this information in breeding research has been lacking. Using data from the Web of Science (WoS) and Orbit Intelligence databases (2013–2023), this study employs the Bibliometrix package in R and Gephi for visual analysis. We examined keywords, co-occurrences, collaborations, and emerging trends to shed light on future research and intellectual property (IP) directions. The findings reveal a consistent rise in annual publications, in contrast to a decline in patent filings. China and the United States emerge as the primary contributors. Analysis of patent records identifies M S Technologies and Monsanto Technology LLC as key players. This study provides a vital reference for researchers and industry stakeholders to address global food security challenges.

## 1. Introduction

Soybean [*Glycine max* (L.) Merrill] is one of the most important legume crops globally and is considered a “golden crop” due to its unique nutritional value. Its seeds are characterized by high oil and protein content, making them a vital source for food, feed, pharmaceuticals, and various industrial materials.^1,2^ Originating in East Asia, the domestication and breeding of this crop have enabled its cultivation worldwide.^3^ North and South America possess the largest cultivated areas, accounting for approximately 80% of global soybean production, with Brazil and the United States leading the sector.^4,5^ Soybean production has steadily increased over the years, reaching approximately 371.7 million tons by 2021 and generating $77,703 million in export revenue, with projections reaching 411 Mt by 2030.^4^ This optimistic outlook supports the needs of a continuously increasing global population, which is projected to grow by nearly 25% by 2050, reaching 9.3 billion people.^6^

Addressing global soybean production demands requires tackling the challenges posed by climate change. Environmental factors, such as fluctuating temperature and precipitation patterns, are predicted to vary more frequently, thereby affecting crop yields.^7^ Significant efforts have been dedicated to advancing soybean research, including the genome sequencing of several varieties, the availability of extensive genomic resources, improved genome editing methods, accurate molecular markers, and advancements in plant transformation and in vitro techniques. These developments collectively provide an extensive array of opportunities for soybean breeding.^8,9^ Among the traits plant breeders strive to improve are biotic and abiotic stress tolerance, nitrogen fixation, and seed quality.^1,9,10^

Consequently, advancements in production practices have led to a remarkable increase in soybean yields in the United States by nearly 30% over the past decade.^11^ A similar trend is evident in southern Brazil’s production over the last 50 years, which has concurrently enhanced stress resistance, oil and protein concentrations, and the number of pods and seeds per plant.^12^ These findings underscore the pivotal role of genetic improvement for major producers and, by extension, for global production.

With the continuous development of new crop varieties and increasing interest in their commercialization, there is a growing need to implement intellectual property (IP) measures to maximize economic benefits.^13^ A patent is a legal IP document that grants an exclusive right for an invention over a predetermined period.^14^ Furthermore, IP activities encompass the protection and exploitation of intellectual assets, though these practices can differ significantly between countries. A genetically modified (GM) plant or event can be protected by multiple patents covering various aspects, such as the DNA sequences used, the event itself, and its specific applications.^15^ Specifically, IP protection grants breeders exclusive rights to new varieties under national and international frameworks, such as the International Union for the Protection of New Varieties of Plants (UPOV) convention.^16^ To address the diversity of patenting models, the World Intellectual Property Organization (WIPO) established the International Patent Classification (IPC). This hierarchical taxonomy serves to streamline the classification of patent documents on a global scale. Currently, numerous patent databases provide extensive information on innovation, categorized by indexes such as publication date, assignees, inventor countries, legal status, and patent classification, often including integrated analytical tools.^17^

Patents and scientific literature are essential for leveraging innovative knowledge and disseminating research activities. Rapid advances in knowledge production have driven the need for analytical measures to evaluate innovation value and its diffusion trajectory.^18^ Bibliometric analysis is an analytical technique increasingly used by the scientific and business communities, allowing for the processing and analysis of massive volumes of data generated from cumulative scientific knowledge. This analysis enables researchers to explore the intellectual structure of a specific area by decoding and mapping trends in scientific production, collaboration patterns, and keyword co-occurrence. As a result, it provides an overview of the field, identifies knowledge gaps, and generates new ideas that contribute to the development of the area.^19,20^

Despite the growing body of scientific literature on soybean biotechnology, there remains a significant gap in the systematic mapping of the intellectual property landscape and its synergy with academic research. To address this, the present study aims to provide a comprehensive bibliometric analysis that integrates both global patenting trends and the metadata of original scientific articles. By leveraging the FamPat database alongside scholarly metadata and network visualization tools, this research characterizes the structural relationships between key jurisdictions, corporate assignees, and technological domains. Furthermore, this study evaluates citation networks, inventor collaborations, and co-authorship patterns that drive the current bioeconomy, offering strategic insights into the concentration of innovation and the evolving trajectory of global soybean research. Ultimately, this dual-source analysis provides a holistic overview of the field, enabling stakeholders to identify emerging technological clusters and bridge the gap between basic scientific discovery and commercial intellectual property protection.

## 2. Materials and Methods

### 2.1. Data Sources and Strategy Analysis

**Figure 1.**
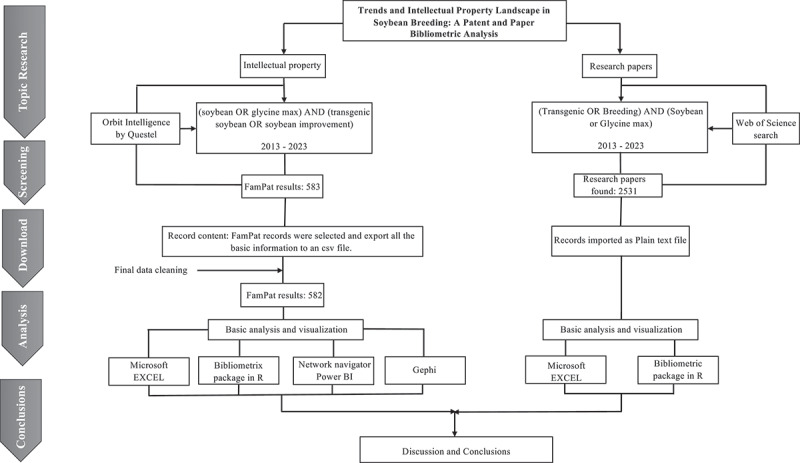
Workflow of the strategy and bibliometric analysis.

The methodological workflow, illustrating the sequence from data acquisition to the integrated analysis of patents and publications, is presented in Fig. 1.

#### 2.1.1. Intellectual Property Data (Orbit Intelligence)

Patent families (FamPat) related to soybean breeding were retrieved from Orbit Intelligence (version 1.9.8) ^21^ on November 1, 2023. An advanced search was conducted using the query: “(soybean OR glycine max) AND (transgenic soybean OR soybean improvement)” within the title, abstract, claims, and object of invention, spanning from January 1, 2013, to November 1, 2023. The exact Boolean search strings, along with the specific filtering parameters used for the initial retrieval, are documented in Supplementary Material S1.

The initial search yielded 1,752 FamPat records. After filtering for “granted/alive” legal status, the dataset was reduced to 1,270 records. To further refine the scope, a technical similarity restriction focused on “transgenic soybean and/or breeding” was applied, resulting in a core dataset of 583 records.

#### 2.1.2. Data Cleaning and Standardization

To ensure the highest data quality, a rigorous manual curation and harmonization process was conducted in MS EXCEL:

1. Disambiguation: Inventor and company names with multiple variations were consolidated by cross-referencing institutional affiliations and co-inventor networks, validated through full-text data from Google Patents.
2. Institutional normalization: Assignees were verified via official corporate websites and investor relations reports. Subsidiaries were grouped under their parent companies (e.g., Pioneer Hi-Bred was grouped under Corteva Agriscience) to reflect current ownership structures.
3. Exclusion criteria: One record (EP2893034 - Method for Multiplex Nucleic Acid Analysis) was excluded as it pertained to broad nucleic acid analysis rather than specific soybean improvement. Consequently, the final analysis was conducted on 582 FamPat records, as detailed in Supplementary Table S1 (Table S1).

Initial trend analysis, encompassing patent volumes, geographical distribution, and technological domains, was performed in MS Excel performed using Excel statistical functions, such as =COUNTIF.

Result trend analyses were processed using the Bibliometrix package (v 4.1.3)^22^ within the R environment (v 4.3.1).^23^ For advanced relational mapping, specific datasets were processed in Gephi (v. 0.10.1).^24^ These visualization tools enabled the examination of linkages between common attributes (targets) and individual FamPat records (sources). For the jurisdiction-based network maps, country names were derived from the ISO two-character codes within the FamPat IDs, and orphan links were removed to ensure network coherence.

Networking analysis was conducted using patent data retrieved from the FamPat worldwide database, focusing on the top 10 most cited patent families (Table 1). Metadata were extracted via Google Patents, applying a strict filter to exclude “cited by examiner (*)” entries to ensure the study prioritized applicant-driven citations. To maintain network integrity and prevent isolated nodes, jurisdictional data from Uruguay (UY) and the World Intellectual Property Organization (WO) were excluded. For the jurisdictional, technological domains, inventors and co-citations mapping, a direct graph configuration was used. The spatial layout was determined by the Fruchterman-Reingold algorithm. Node label sizes were scaled according to degree centrality to identify influential hubs, while Modularity-Based Clustering (MBC) was utilized to categorize distinct neighborhoods.

**Table 1.** Top 10 alive and granted FamPat, with the ranking based on the number of full citing patents (cp).

Rank	ID	Title	Institution	CP
1	EP2892321	Fad2 performance loci and corresponding target site specific binding proteins capable of inducing targeted breaks	Sangamo Therapeutics Inc, Corteva Agriscience LLC, Dow Agrosciences	460
2	EP2736321	Insect resistant and herbicide tolerant soybean event 9582.814.19.1	Corteva Agriscience LLC, Dow Agrosciences, Kedihua Agricultural Technology	217
3	EP3008187	Soybean transgenic event mon87751 and methods for detection and use thereof	Monsanto Technology	163
4	EP2731419	Stacked herbicide tolerance event 8264.42.32.1, related transgenic soybean lines, and detection thereof	Dow AgroSciences, M S Technologies LLC	144
5	EP3207049	Novel chimeric insecticidal proteins toxic or inhibitory to lepidopteran pests	Monsanto Technology	85
6	EP3066109	Optimal soybean loci	Corteva Agriscience LLC, Dow AgroSciences	84
7	EP3008187	Soybean transgenic event mon87751 and methods for detection and use thereof	Monsanto Technology	76
8	EP3677669	Production of dha and other lc-pufas in plants	Corteva Agriscience LLC, Dow Agrosciences, DSM IP Assets BV	62
9	US10900052	Compositions and methods for increasing nematode resistance in plants	Syngenta Participations AG, Evogene Ltd	56
10	EP2736917	Soybean event pdab9582.814.19.1 detection method	Corteva Agriscience LLC, Dow Agrosciences, Kedihua Agricultural Technology	54

Furthermore, citation network maps were generated for the top 10 most cited patent families from the FamPat database (Table 1) to visualize inter-firm knowledge flows. Within these maps, the width of the directional arrows (edges) represents the citation weight between major biotechnology entities. To enhance the clarity of the primary connections, a weight filter greater than 3.0 was applied, and the preview ratio was adjusted to 30% to highlight the major assignees within each cluster and their respective self-citations.

#### 2.1.3. Research Papers

The research paper records were obtained on November 01, 2023 from the Web of Science database using the following Boolean query: (Transgenic OR Breeding) AND (Soybean or Glycine max). We filtered the results by topic, language (English) and time span (2013 to 2023), and excluded review papers. The search records were imported as plain text file and analyzed to determine production of research papers over the years, the main contributors, the most important themes and how they interact. The data were processed, and the results extracted using the Bibliometrix package (version 4.1.3)^22^ on R (version 4.3.1)^23^ (Fig. 1).

## 3. Results

### 3.1. Patent Analysis on Soybean Breeding Over the Last Decade

#### 3.1.1. Basic Overview of Inventions

**Figure 2.**
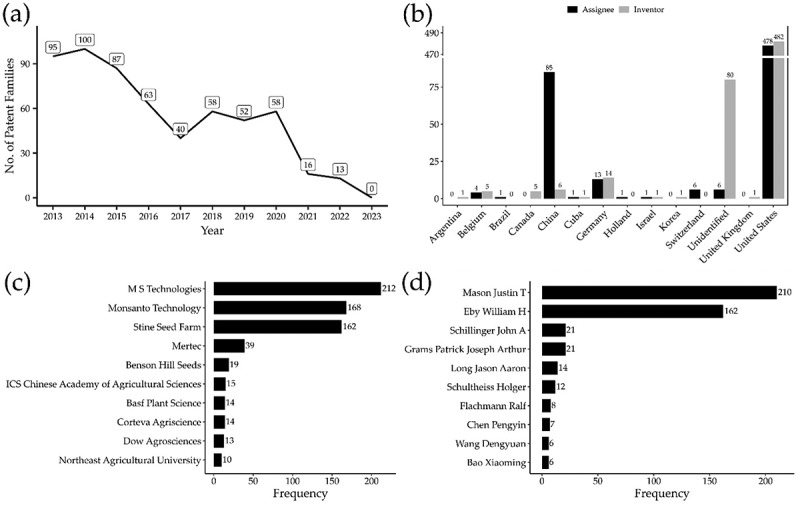
Patent family landscape in soybean breeding. (a) Annual distribution and trends of patent families from 2013 to 2023. (b) Top 10 countries by number of assignees and inventors. (c) Top 10 institutions and companies by number of patent families. (d) Top 10 most prolific inventors by FamPat.

The trend of patent families shows a significant surge in 2014, followed by a steady decline with modest recoveries between 2018 and 2020 (Fig. 2a). Analysis of the 582 FamPat instances identifies the U.S. as the dominant leader, accounting for over 80% of both inventors and assignees. While Germany (2.34%) and China (1.0%) follow in inventor counts, the assignee landscape reveals that China rises to second place with 14.3% of patent ownership (Fig. 2b).

**Figure 3.**
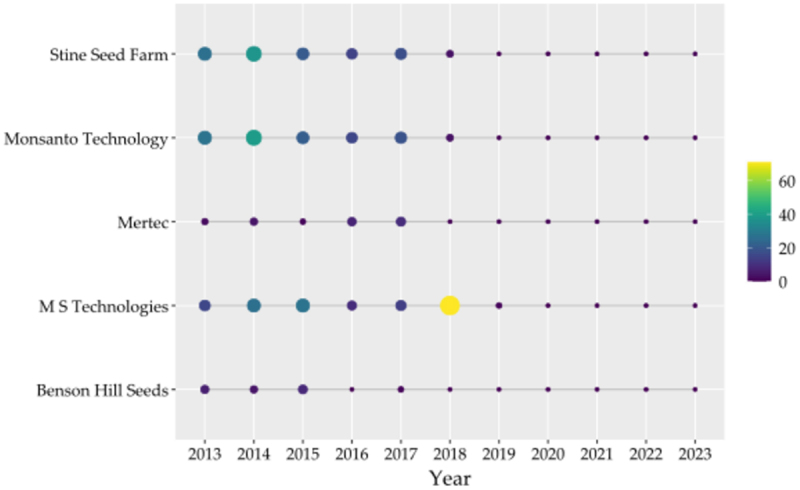
Temporal evolution of patent filings for the top five institutions (2013–2023). Each circle represents the annual volume of patent applications submitted by these institutions. The size of the circles is proportional to the total number of patent families, while the color intensity reflects the concentration of granted patents.

MS Technologies leads in active and granted FamPat instances, closely followed by Monsanto Technology LLC and Stine Seed Farm (Fig. 2c), with primary inventors Justin T. Mason and William H. Eby contributing over 60% of total patented inventions (Fig. 2d). Chronologically, these three institutions dominated the first six years of the decade, though MS Technologies specifically distinguished itself as the top contributor in 2018 (Fig. 3). Finally, high-impact intellectual property is concentrated within Corteva Agriscience LLC and Dow AgroSciences LLC, which hold the ten most-cited patent families, with citation counts reaching as high as 460 (Table 1).

In summary, the data reflects a highly concentrated patent landscape dominated by U.S.-based institutions and a few prolific inventors, with a recent alteration toward increased Chinese institutional ownership.

**Figure 4.**
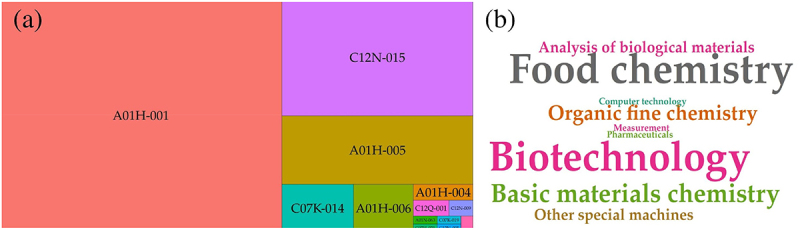
Treemap of top 10 main international patents. For the three main codes see the text below. C07K-014/ 019 (chemical organic- peptides/Hybrid peptides); A01H-006/004 (plant reproduction by tissue culture technique/Angiosperms – flowering plants); C12Q-001 (measuring or testing process involving enzymes, nucleic acids or microorganisms); C12N-009/005 (genetic mutation or engineering of enzymes/Undifferentiated plant cells or cell lines); A01N-063 (plant preservation using biocides, plant growth regulators); C07H-21 (sugar, compounds containing two or more mononucleotide units having separate phosphate or polyphosphate groups linked by saccharide radicals of nucleoside groups). (b) Word cloud of patent technology domains. Word size reflects the frequency of occurrence (*N* = 582 FamPat), providing a quick interpretation of the most prominent domains.

Regarding the main IPC for utility patents (Fig. 4a), the most frequently used category was A01H-001, which refers to processes for modifying plant genotypes, particularly within the field of reproduction by tissue culture techniques for agriculture. The second most frequent category was C12N-015, pertaining to the manipulation of vectors, genetic material, and the use of hosts for genetic engineering goals. A01H-005 was the third most used category, covering processes involved in the production of hybrid plants using tissue culture techniques.^25^ Complementing these classifications, Figure 4b illustrates that the predominant technology domains associated with this intellectual property were “food chemistry” and “biotechnology,” respectively. Other notable domains included “computer technology,” “pharmaceuticals,” and ‘measurement.

#### 3.1.2. Network Analysis

**Figure 5.**
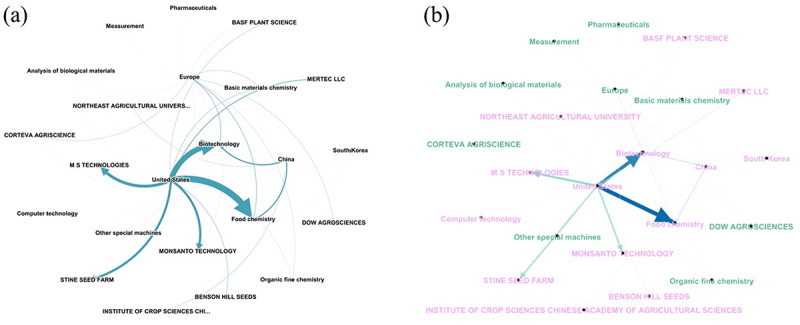
Networking map of jurisdiction, corporate assignees, and technological domains. Nodes represent geographic origins (e.g., China, United States), patent assignments (e.g., Monsanto, BASF), and IPC/Tech domains (e.g., biotechnology, food Chemistry). a) dark-themed visualization highlighting global connectivity; b) the network topology displays the centrality of the ‘United States’ (light pink) and ‘europe’ (light green) as bridges between various agricultural companies and technology domains. The arrows indicate the direction of intellectual property flow from jurisdiction toward TechDom and assignments. Edge thickness is proportional to the number of related fampat entries, with normalized weights ranging from 0.1 to 1.0.

The network map of the top 10 assignee jurisdictions and technology domains reveals that the majority of FamPat instances originate from the U.S., held primarily by three leading biotechnology players: MS Technologies, Monsanto Technology LLC, and Stine Seed Farm. The network edges illustrate the direction of intellectual property flow from these jurisdictions toward specific technological domains, while node label sizes-scaled by degree centrality-highlight “Food Chemistry” and “Biotechnology” as the primary areas of convergence (Fig. 5a). In contrast, European FamPat instances exhibit a broader and more diversified distribution across domains such as “Organic Fine Chemistry,” and “Basic Materials Chemistry” primarily under the protection of Corteva Agriscience and Dow Agrosciences (Fig. 5b). This structural disparity suggests that while the U.S. landscape is characterized by a high concentration of specialized corporate leadership, the European sector maintains a more heterogeneous technological footprint.

**Figure 6.**
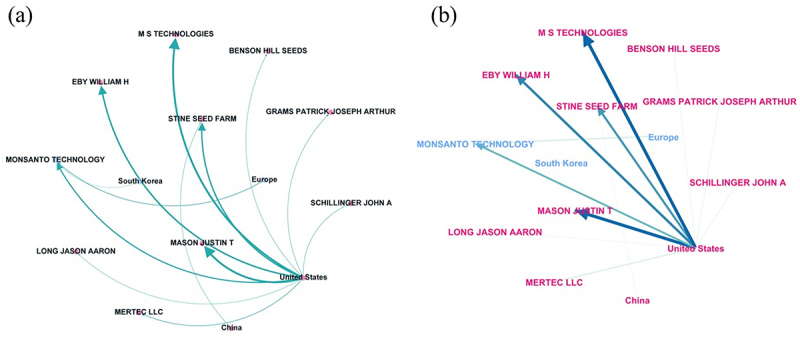
Networking map illustrating the occurrence of FamPat categorized by the top 5 assigned institutions and inventors. Nodes represent geographic origins (e.g., China, United States), patent assignments (e.g., Monsanto, BASF), and inventors associated (e.g., Mason Justin t, Eby William H). a) dark-themed visualization highlighting global connectivity; b) the network topology displays the centrality of the ‘United States’ (light pink) and ‘europe’ (light blue) as bridges between various agricultural companies and inventors. The arrows indicate the direction of intellectual property flow from jurisdiction toward assignments and inventors. Edge thickness is proportional to the number of related fampat entries, with normalized weights ranging from 0.1 to 1.0.

In the network analysis of the top five intellectual property players, MS Technologies and Monsanto Technology LLC are directly linked to key inventors William H. Eby and Justin T. Mason. Notably, both inventors have filed patents across multiple institutions; for instance, the lead inventor, Justin T. Mason, has patented with both MS Technologies and Mertec LLC. Similarly, William H. Eby is associated with both Monsanto Technology LLC and Stine Seed Farm. Furthermore, the majority of FamPat instances related to these core institutions and inventors are concentrated within the United States jurisdiction (Fig. 6).

**Figure 7.**
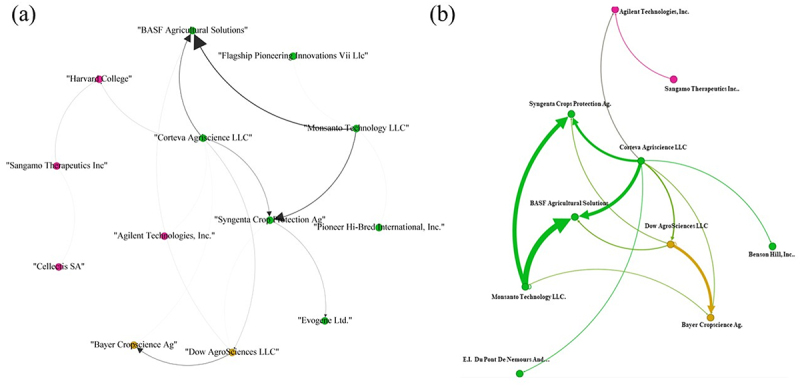
Patent citation network maps based on the top 10 citing patents (cp) from the FamPat database (Table 1). a) network visualization of citations for the main assignees. b) the map displays nodes with a weight greater than 3 (range: 1.0–153.0). b) network map highlighting key assignees and their citation collaborations. Clusters are color-coded as follows: green (Monsanto Technology LLC), yellow (Dow AgroSciences LLC), and Pink (Sangamo Therapeutics Inc.). Edge thickness is proportional to the number of cited patents, with normalized weights ranging from 0.1 to 1.0.

Based on the ten most-cited FamPat instances (Table 1), the citation network map reveals three primary clusters formed among prominent biotechnology entities, including Monsanto Technology LLC, Sangamo Therapeutics Inc., and Dow AgroSciences LLC (Fig. 7). The green cluster illustrates the network between major industry players, where Monsanto Technology LLC and Corteva Agriscience LLC received the highest citation volumes, primarily from Syngenta Crop Protection AG and BASF Agricultural Solutions.

Additionally, the yellow cluster shows that Bayer CropScience AG and Pairwise Plants Services, Inc. account for the majority of citations directed toward Dow AgroSciences LLC’s FamPat records. Finally, the pink cluster centers on Sangamo Therapeutics Inc., containing several FamPat records cited individually by various distinct entities. A detailed view of this network (Fig. 7b) highlights two main clusters exhibiting significant self-citations by Monsanto Technology LLC and Dow AgroSciences LLC, where citation weight is visually represented by the thickness of the directional arrows between nodes.

### 3.2. Research Papers Over the Last Decade

#### 3.2.1. Basic Overview Analysis

**Figure 8.**
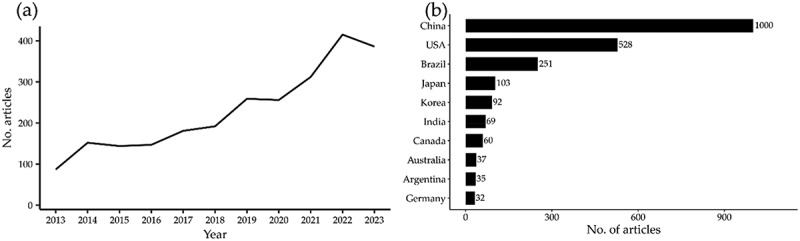
Bibliometric analysis of publications (2013–2023). Line graph (a) demonstrates the annual growth trend, while bar chart (b) presents the distribution of academic production by country of origin.

Our search in the Web of Science (WoS) returned 2,531 documents distributed across 475 journals. The number of papers published increased steadily from 2013 to 2022, representing an increase of 377%, with a slight decrease in 2023 that could be attributed to the search cut date of November 1st (Fig. 8a). Most publications originated from China (39.4%), followed by the U.S. (20.8%), Brazil (9.8%), Japan (4%) and Korea (3.6%) (Fig. 8b). These five countries were responsible for 77.7% of the global publications. However, we identified publications from 72 other countries.

**Figure 9.**
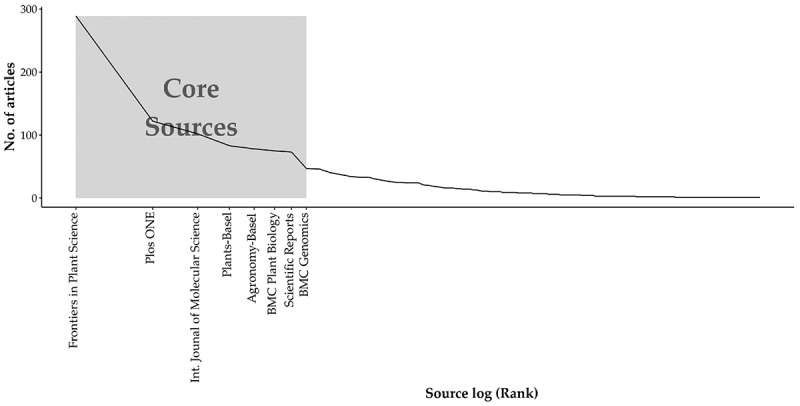
Bradford’s law graphics. The graphic shows how the archives are distributed across the most relevant journals (core sources).

To identify the most relevant sources from our initial search, we applied Bradford’s law analysis, which describes how articles are distributed across journals. The Core Sources corresponds to the journals that published most relevant articles of our theme. In this case, our Core Sources (*n* = 8) (Fig. 9) published 34.3% of all articles.

**Figure 10.**
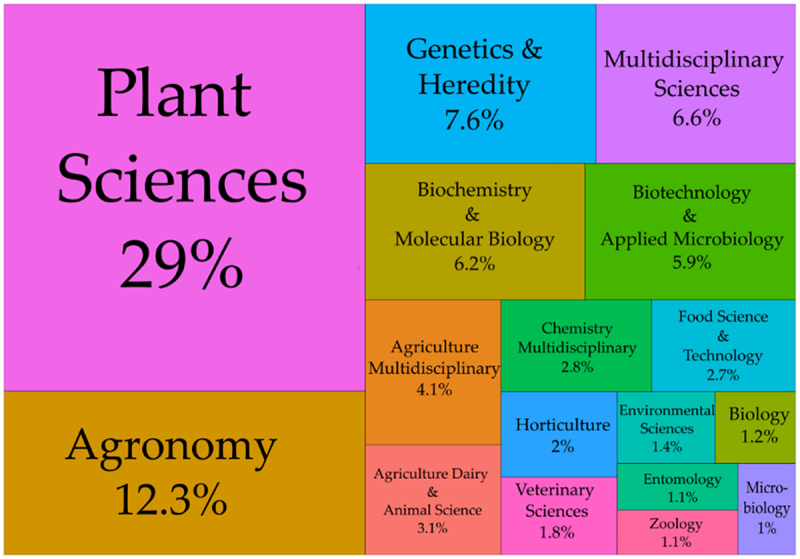
Distribution of original papers by research area. The treemap illustrates the thematic classification of publications according to journal categories. The size of each block is proportional to its percentage share, with plant sciences (29%) and agronomy (12.3%) representing the most prominent fields.

In the WoS platform, journals are classified in general categories, and we found 50 different categories. About 80% of the papers were classified under 10 categories, which are related to plant science, agriculture, and molecular biology (Fig. 10). Some categories that appeared in lower frequency (e.g., fisheries, geology, oncology) are beyond the scope of our work and are not presented in Figure 9 (see the full list of categories in Supplementary Table S2 (Table S2). Hereafter, we will focus on the most representative categories.

#### 3.2.2. Keyword Analysis

**Figure 11.**
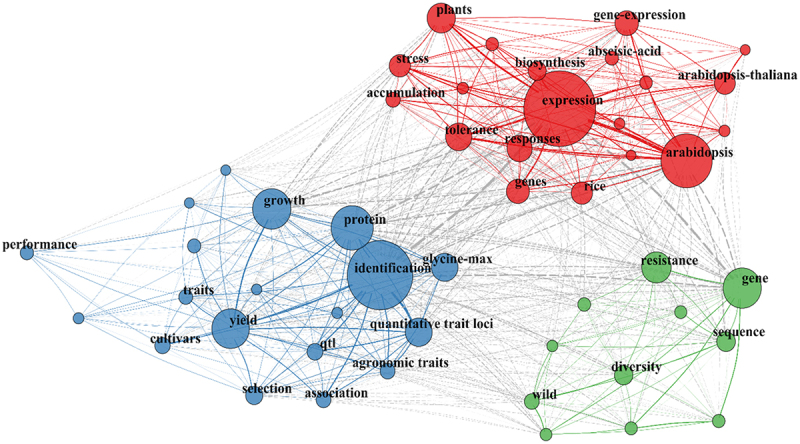
Keyword co-occurrence network illustrating the thematic structure of the research field. Nodes represent the most frequent terms, with size proportional to their occurrence, while edges indicate the strength of association between them. Three main clusters can be observed: in green, related to genetic diversity, selection, and resistance; in blue, associated with agronomic traits, yield, and QTL identification; and in red, linked to gene expression, stress, and physiological responses in plants.

A total of 5,896 keywords were identified. To facilitate visualization and evaluate the most relevant terms, a co-occurrence network analysis was performed using the 50 most frequent keywords (Fig. 11). The network formed three distinct clusters centered around the terms “expression,” “identification,” and “gene.” Based on the thematic analysis of the articles within each group, the clusters were categorized as follows: Cluster 1 (blue), related to agronomic traits and yield; Cluster 2 (red), focusing on molecular mechanisms and model plants; and Cluster 3 (green), pertaining to genetic diversity and genomics. In this network, the betweenness centrality values highlight the keywords that act as primary bridges between research topics, identifying them as central pillars of soybean breeding research over the last decade.

#### 3.2.3. Author Contributions

**Figure 12.**
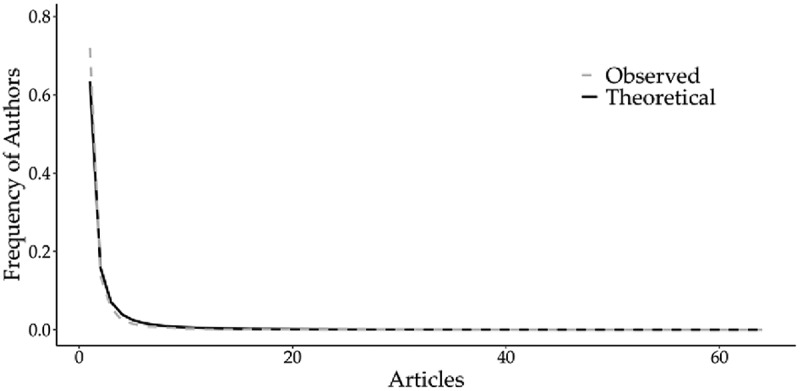
Author productivity and Lotka’s law distribution. The graph compares the theoretical distribution of Lotka’s law (theoretical line) against the empirical distribution of the collected data (observed line).

**Figure 13.**
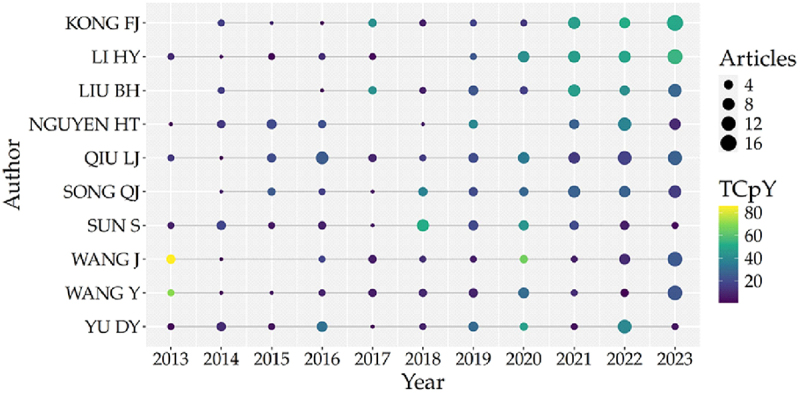
Top 10 authors’ production over time in soybean breeding (2013–2023). The timeline illustrates the publication consistency and impact of the most prolific authors. The size of the circles is proportional to the number of articles published per year, while color intensity represents total citations per year (TCpY), with yellow indicating higher citation impact.

The search identified a total of 10,403 authors. Of these, 95% published fewer than five papers, while the top ten authors contributed at least 39 papers each (Fig. 12), aligning with Lotka’s Law. This law, which describes author productivity, posits that a small number of prolific authors produce the majority of publications in a given scientific field. Furthermore, most of these leading authors have remained active in soybean breeding research since 2013, with a notable increase in publication volume over the last three years (2021–2023) (Fig. 13).

**Figure 14.**
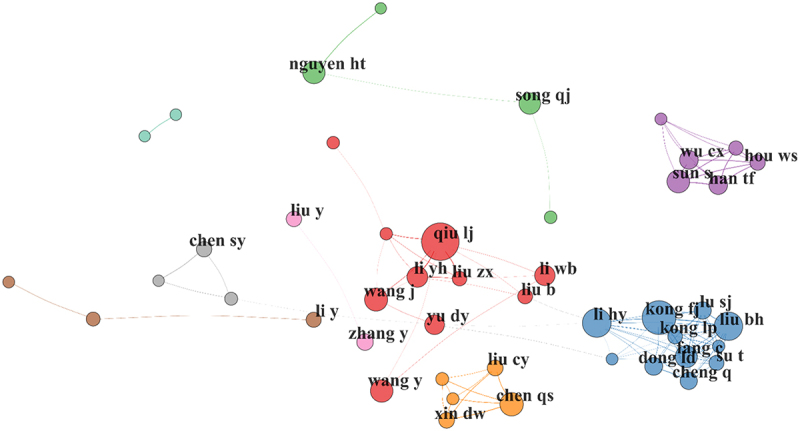
Co-authorship network illustrating the collaboration structure among authors in soybean breeding research. Nodes represent authors, with size proportional to their scientific production, while edges indicate collaborative relationships. Colors denote distinct collaboration clusters, highlighting research groups and their interaction patterns within the field.

A collaboration network analysis was conducted on the most productive 5% of authors (520 in total). The analysis revealed 55 distinct clusters, the majority of which were small and isolated from the primary network. The principal clusters are led by authors with high h- and g-indices (Fig. 14 and Table 2). Furthermore, authors were ranked by local citations (LC), a metric reflecting the frequency of citations an author received within the specific document set analyzed for this study.

**Table 2.** Bibliometric indicators of the most productive authors in soybean breeding research. The table presents the h-index, g-index, total citations (TC), number of publications (NP), and local citations (LC) for leading authors. The metrics highlight differences in scientific productivity and impact, with some authors showing high publication output, while others demonstrate greater citation influence within the field.

Author		*h*-index	*g*-index	TC	NP	Author	LC	*h*-index	*g*-index	TC	NP
Wang J	15		40	1904	40	Song QJ	351	19	35	1282	43
Wang Y	15		38	1470	40	Fang C	310	13	28	1647	28
Kong FJ	19		37	1388	44	Kong FJ	287	19	37	1388	44
Sun S	20		36	1349	39	Qiu LJ	283	21	34	1320	64
Song QJ	19		35	1282	43	Liu BH	273	18	35	1261	40
Liu BH	18		35	1261	40	Sun S	267	20	36	1349	39
Qiu LJ	21		34	1320	64	Wu CX	257	19	32	1240	32
Li HY	21		33	1214	52	Chen SY	243	20	26	1362	26
Yu DY	19		33	1156	40	Han TF	240	18	30	1173	30
Nguyen HT	21		33	1149	39	Hou WS	222	17	27	1097	27
Wu CX	19		32	1240	32	Jiang BJ	206	16	25	1010	25
Li WB	20		31	1023	38	Nguyen HT	197	21	33	1149	39
Han TF	18		30	1173	30	Wang J	183	15	40	1904	40
Fang C	13		28	1647	28	Yu DY	183	19	33	1156	40
Gai JY	14		27	750	33	Li WB	182	20	31	1023	38
Hou WS	17		27	1097	27	Li HY	159	21	33	1214	52
Li YH	14		26	698	32	Cheng Q	156	19	24	1006	24
Chen SY	20		26	1362	26	Dong LD	152	18	26	974	26
Dong LD	18		26	974	26	Zhao TJ	139	15	24	594	29
Liu Y	13		25	626	28	Lu SJ	139	12	21	626	21
Jiang BJ	16		25	1010	25	Li YH	138	14	26	698	32
Li Y	13		24	600	30	Chen L	135	12	22	919	22
Zhao TJ	15		24	594	29	Gai JY	126	14	27	750	33
Nian H	14		24	628	28	Wang Y	124	15	38	1470	40

#### 3.2.4. Countries Collaboration Network

**Figure 15.**
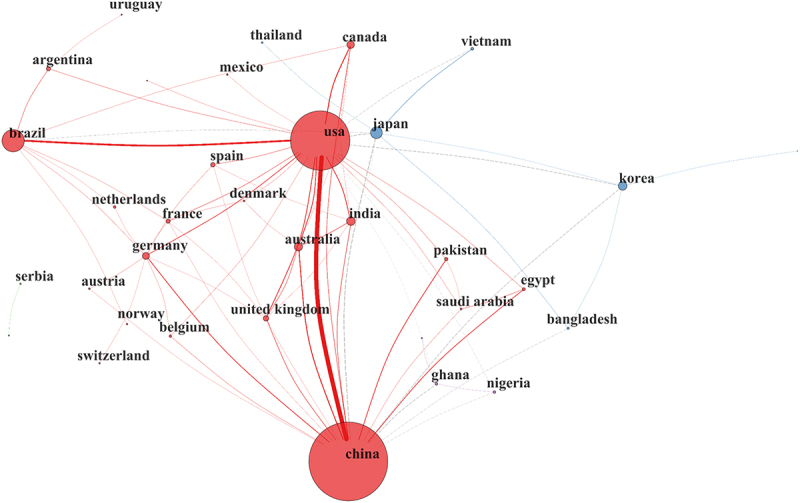
International collaboration network in soybean breeding research. Nodes represent countries, with size proportional to their scientific output, while edges indicate collaborative links between them, with thickness reflecting the strength of collaboration. The network highlights the central role of countries such as the U.S., China and Brazil, which exhibit the highest connectivity and act as major hubs in global research collaboration.

A collaboration network analysis was performed on the 72 identified countries, resulting in seven distinct clusters (Fig. 15). Clusters 3 and 4 emerged as the most prominent. While Cluster 3 consists primarily of countries with lower research output, Cluster 4 (red) represents the most influential nodes in the network, led by the U.S., China, and Brazil. These three nations exhibit the highest collaboration density, with the strongest link observed between the U.S. and China. Notably, European countries within this same cluster, such as Germany (*n* = 32), Belgium (*n* = 11), and Norway (*n* = 8), act as key collaborative partners despite their relatively lower individual production, highlighting a highly integrated transatlantic and transpacific research axis.

**Figure 16.**
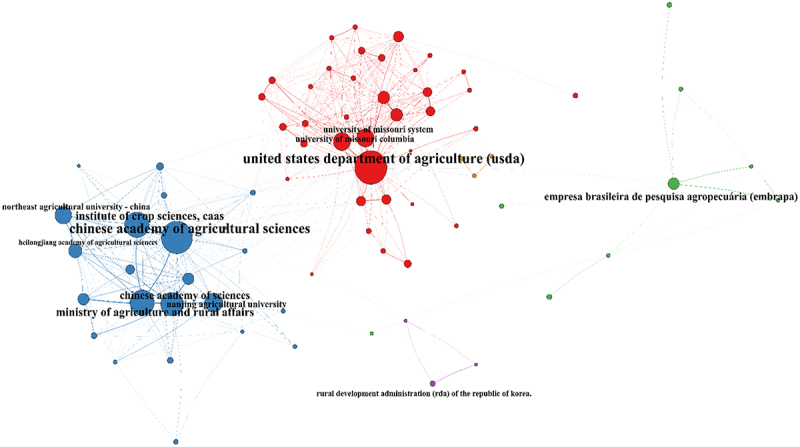
Institutional collaboration network in soybean breeding research. Nodes represent research institutions, with their size proportional to the total number of publications. The lines (edges) indicate the strength of collaborative links. Three dominant clusters are identified: the North American cluster (red), led by the USDA and the University of Missouri; the Chinese cluster (blue), centered around the Chinese Academy of agricultural sciences (CAAS); and the South American cluster (green), led by Embrapa.

The institution collaboration network formed 4 clusters (Fig. 16), led by China (blue); U.S. (red); Brazil (green) and the Republic of Korea (purple). China and the U.S. had the largest number of institutions and collaborations, and most of these collaborations were domestic. The most prominent institutions in each cluster were the Chinese Academy of Sciences, the United States Department of Agriculture (USDA), the Empresa Brasileira de Pesquisa Agropecuária (EMBRAPA) from Brazil, and the Rural Development Administration (RDA) of the Republic of Korea.

## 4. Discussion

The longitudinal analysis of the Orbit Intelligence database reveals a significant decline in patent activity related to soybean breeding following its peak in 2014. This trend suggests that the early 2010s represented a critical transition for the industry. A primary catalyst for this surge and subsequent decline was the imminent expiration of the first major commercial biotech gene patent in the U.S.: Monsanto’s Roundup Ready soybean event (GTS 40–3-2), which at the time was adopted by over 90% of U.S. soybean farmers. This expiration generated substantial uncertainty for farmers and traders while reducing financial incentives for original developers.^26^ In response, the AgAccord framework was established to manage off-patent events through the “Generic Event Marketability and Access Agreement (GEMAA)” and the “Data Use and Compensation Agreement (DUCA).”^27^ This framework allowed off-patent events to be repurposed by other institutions to develop new beneficial varieties.^15^

However, the post-2014 decline is a multifaceted phenomenon. Beyond patent expirations, broader industry consolidation played a pivotal role; a wave of mega-mergers (notably the formation of Bayer-Monsanto, Corteva (Dow-Dupont), and Syngenta -ChemChina) consolidated the “Big Six” into a “Big Four.”^28^ This consolidation led to the streamlining of R&D pipelines, where companies prioritized high-value “blockbuster” traits over numerous incremental filings. Furthermore, regulatory shifts in global markets have increased the complexity and cost of transgenic event approval, while a strategic shift in Intellectual Property (IP) protection has led firms to increasingly utilize Plant Variety Protection (PVP) and trade secrets as complementary tools to safeguard germplasm.^29^ Finally, the apparent decline observed in the most recent years of the dataset (2021–2023) is likely attributed to the standard 18-month publication lag and the extended 3–5 year examination period typical of major intellectual property offices, rather than a recent decline in innovation activity.^30,31^

In terms of institutional contributions, the U.S. remains the dominant force in IP, accounting for 81% of the analyzed records. This dominance is sustained by state-of-the-art research centers and a regulatory environment that fosters IP growth.^32^ Major biotechnology consortiums, such as the collaboration between Bayer CropSciences, Mertec LLC, and M S Technologies, have become essential to sustain these high-cost IP activities.^33^ Our network analysis underscores this robust collaboration, evidenced by shared inventors like Justin T. Mason and William H. Eby, who bridge multiple “seed giants” to maintain control over global genetic resources.

A notable finding in our analysis is that China holds 14.3% of the identified patents despite accounting for only 1.0% of the individual inventors. This marked discrepancy suggests that Chinese firms and institutions may be aggressively acquiring or licensing foreign IP and portfolios to bolster their domestic biotech sector, rather than relying solely on internally generated inventions. This strategy aligns with China’s national strategic mandate to rapidly enhance food security and reduce its heavy reliance on soybean imports.^5,34,35^ This dichotomy defines the current landscape: a private sector focused on commercial IP in the West and a public sector focused on academic knowledge expansion in Asia.

Thematic analysis reveals that IP sector prioritizes “Food Chemistry” and “Biotechnology.” The term “food chemistry” encompasses the characterization of compounds, food conservation and transformation treatments, the modernization of food technology, and studies on the extraction of valuable resources from food plants.^36^ The high citation count for patents like EP2892321 (FAD2 gene modification) highlights a shift toward high-value nutritional traits, such as modifying oleic acid content, which is crucial for human health.^37,38^ Academic research, meanwhile, centers on functional biology, gene expression, and yield trait identification, reflecting a strategic interest in functional molecular biology as a pivotal tool for crop improvement.^39,40^

Currently, the intricate connection between global agriculture and the food supply faces challenges that threaten worldwide food security. GM crops are regarded as a robust solution, as they can be developed to achieve higher yields, resistance to biotic and abiotic stress, and improved nutritional quality.^41,42^ Nevertheless, as noted in previous studies, some GM crops are still perceived as a risk to human health, despite a dearth of solid scientific evidence to support such concerns.^41^ Despite these public apprehensions, the industry continues to grow, emphasizing the discovery of new gene banks and molecular tools. Major companies are increasingly focused on creating new varieties primarily through trait crossings and are likely to collaborate with smaller biotechnology firms. This strategic collaboration aims to reduce IP maintenance costs and provide the necessary economic support for innovative solutions. Ultimately, despite persistent public concerns regarding Genetically Modified (GM) crops, the robust connection between the U.S. and China suggests that scientific cooperation remains a strategic necessity for global food security.

## 5. Conclusions

This study provides a comprehensive mapping of the soybean breeding landscape over the last decade. Our findings reveal a shift from an IP-centric surge around 2014, driven by the expiration of foundational patents and industry consolidation, toward a more diversified scientific expansion led by Chinese academic institutions. While the United States continues to lead in private-sector innovation and intellectual property through major seed consortiums, China has emerged as the global leader in scientific publication volume, motivated by national food security strategies.

The integration of patent and bibliometric data highlights a clear thematic trajectory: the industry is moving basic yield improvement toward complex molecular trait modification, such as fatty acid desaturation and abiotic stress tolerance. Furthermore, the robust collaboration networks identified, particularly the U.S-China research axis, suggest that the future of soybean advancement depends on highly integrated global cooperation. This work serves as a strategic roadmap for researchers and inventors, identifying the key players and technological shifts that will define the next decade of soybean breeding and its vital role in global food security.

## Supplementary Material

Supplementary tables.xlsx

Supplementary Material S1.docx

## Data Availability

The data utilized in this research is available within the article and its supplementary materials.
