# Supplementary material for: Trends and intellectual property landscape in soybean breeding: a patent and paper Bibliometric analysis
Source: GM Crops Food. 2026 May 5;17(1):2662120. doi: 10.1080/21645698.2026.2662120 (PMC13154963; doi:10.1080/21645698.2026.2662120)
Supplement: Supplementary Material S1.docx [file KGMC_A_2662120_SM6413.docx]

**Supplementary Material S1**

1. **Patent Data Retrieval (Orbit Intelligence)**

The patent search was conducted using the **FamPat database** (Orbit Intelligence) to retrieve unique patent families. The selection followed a rigorous four-stage filtering process:

- **Database:** Orbit Intelligence (Questel).
- **Search Scope:** Title, Abstract, Claims, and Object of Invention (/TI/AB/CLMS/OBJ).
- **Boolean Query:** (soybean OR glycine max) AND (transgenic soybean OR soybean improves)
- **Timeframe:** First publication date between January 1, 2013, and November 1, 2023.
- **Initial Results:** 1,752 Patent Families (FamPat).

1. **Data Filtering Pipeline:**

To ensure the high relevance of the records, the following screenings were applied:

- **Primary Screening (Legal Status):** Limited to "Alive" patents (including both Granted and Pending applications), resulting in **1,395 FamPat**.
- **Secondary Screening (Legal Status):** Focused on "Granted" patents to ensure the technical maturity of the analyzed inventions, resulting in **1,270 FamPat**.
- **Tertiary Screening (Technical Relevance):** A manual restriction was applied based on string similarity and semantic relevance to **"Transgenic soybean"** within the title, abstract, and claims. This final curation resulted in a refined dataset of **583 FamPat**.

## Data Export and Metadata Extraction

Once the tertiary screening was completed, the final dataset of **583 FamPat** records was exported from Orbit Intelligence in both **.XLSX (Excel)**. To ensure a multidimensional analysis, the following metadata fields were extracted for each patent family:

- **Temporal Data:** Earliest application date and standardized publication numbers.
- **Actor Metadata:** Inventors, Current Assignees, and Assignee-Inventor country of origin.
- **Technical Content:** Titles, Key words in context, Technical concepts, and Technology domains.
- **Classification:** Main International Patent Classification (IPC) codes.
- **Citation Network Data:** All cited patent numbers (backward citations) and all citing patent numbers (forward citations).

This comprehensive dataset provided the foundation for the subsequent mapping of technological trajectories, geographical distribution, and the identification of "blockbuster" patents through citation analysis.

1. **Data Cleaning and Curation Protocol**

- **Final Manual Curation:** During the deep cleaning phase, a key record regarding *"Method for Multiplex Nucleic Acid Analysis"* was excluded. Although it appeared in the initial search, its scope pertained to broad mutation detection across various organisms rather than specific soybean improvement.
- **Final Dataset:** The analysis was conducted on **582 FamPat records**, as detailed in **Supplementary Table 1 (Table S1)**.

Initial trend analysis of the 582 records was conducted using Microsoft Excel, covering patent volumes, geographical distribution, and technological domains. Subsequently, a manual curation was performed to eliminate ambiguities and ensure data integrity:

## A. Author and Inventor Disambiguation

Inventors with multiple name variations (e.g., 'Mason, J.' vs. 'Mason, Justin T.') were consolidated by cross-referencing institutional affiliations and co-inventor networks. This disambiguation process was further compared and validated by consulting detailed records from Google Patents.

## B. Assignee and Corporate Normalization

To ensure the accuracy of the institutional data, corporate assignees were manually verified. This process involved consulting official corporate websites and investor relations reports to confirm the current legal status and ownership of the firms involved in soybean breeding. This manual validation was essential to identify the key players in the sector and was subsequently used as the basis for the strategic analysis of Mergers and Acquisitions (M&A) presented in the Discussion section.

## Data Processing

## Statistical counting functions (=COUNTIF) were employed to determine the frequency and distribution of key variables, including geographical origin, inventor productivity, technological domains, and corporate assignees. This stage allowed for a foundational mapping of patent volumes before advanced visualization.
